# Robotic-Assisted Pelvic Exenteration for Cervical Cancer: A Systematic Review and Novel Insights into Compartment-Based Imaging

**DOI:** 10.3390/jcm13133673

**Published:** 2024-06-24

**Authors:** Philippe Van Trappen, Marie-Sofie Walgraeve, Sarah Roels, Nele Claes, Eveline De Cuypere, Frederic Baekelandt, Harm Arentsen

**Affiliations:** 1 Department of Gynecological Oncology, AZ Sint-Jan Hospital Bruges, Ruddershove 10, 8000 Bruges, Belgium; 2 Department of Radiology, AZ Sint-Jan Hospital Bruges, Ruddershove 10, 8000 Bruges, Belgium; marie-sofie.walgraeve@azsintjan.be; 3 Department of Radiation Oncology, AZ Sint-Jan Hospital Bruges, Ruddershove 10, 8000 Bruges, Belgium; sarah.roels@azsintjan.be; 4 Department of Medical Oncology, AZ Sint-Jan Hospital Bruges, Ruddershove 10, 8000 Bruges, Belgium; nele.claes@azsintjan.be (N.C.); eveline.decuypere@azsintjan.be (E.D.C.); 5 Department of Urology, AZ Sint-Lucas Hospital Bruges, Sint-Lucaslaan 29, 8310 Bruges, Belgium; frederic.baekelandt@stlucas.be; 6 Department of Urology, AZ Sint-Jan Hospital Bruges, Ruddershove 10, 8000 Bruges, Belgium; harm.arentsen@azsintjan.be

**Keywords:** cervical cancer, compartment-based, exenteration, magnetic resonance imaging, pelvic exenteration, recurrent disease, robotic

## Abstract

**Background:** Patients with persistent or recurrent cervical cancer, following primary treatment with concurrent chemoradiation, represent a subgroup eligible for pelvic exenteration. In light of the substantial morbidity associated with open pelvic exenterations, minimally invasive surgical techniques have been introduced. This systematic review aims to analyze and discuss the current literature on robotic-assisted pelvic exenterations in cervical cancer. In addition, novel aspects of compartment-based magnetic resonance imaging (MRI) are highlighted. **Methods:** This systematic review followed the PRISMA guidelines, and a comprehensive literature search on robotic-assisted pelvic exenterations in cervical cancer was conducted to assess, as main objectives, early and late postoperative complications as well as oncological outcomes. Inclusion and exclusion criteria were applied to select eligible studies. **Results:** Among the reported cases of robotic-assisted pelvic exenterations in cervical cancer, 79.4% are anterior pelvic exenterations. Intraoperative complications are minimal and early/late major complications averaged between 30–35%, which is lower compared to open pelvic exenterations. Oncological outcomes are similar between robotic and open pelvic exenterations. Sensitivity for locoregional invasion increases up to 93% for compartment-based MRI in colorectal cancer. A refined delineation of the seven pelvic compartments for cervical cancer is proposed here. **Conclusions:** Robotic-assisted pelvic exenterations have demonstrated feasibility and safety, with reduced rates of major complications compared to open surgery, while maintaining surgical efficiency and oncological outcomes. Compartment-based MRI holds promise for standardizing the selection and categorization of pelvic exenteration procedures.

## 1. Introduction 

Globally, cervical cancer ranks as the fourth most prevalent and fatal cancer among women, as evidenced by 604,000 new cases and 342,000 fatalities reported in 2020 [1]. The age demographic between 40 and 50 years exhibits the highest incidence of cervical cancer, accounting for roughly 20 cases per 100,000 women.

The primary treatment for early-stage cervical cancer is surgery and ranges from conization for stage IA1 to simple/radical hysterectomy with pelvic sentinel lymph node biopsy/lymphadenectomy for stages IA2-IB2 and IIA1. The type of hysterectomy depends on the presence of prognostic risk factors on preoperative imaging and biopsy/conization including the size and stromal invasion of the tumor [2,3,4]. The recently published multicenter randomized SHAPE trial showed similar recurrence rates, at 3 years follow-up, between radical and simple hysterectomy for low-risk cervical cancer, with recurrence rates of 2.17 and 2.52%, respectively [3]. The cervical tumors included were less than 2 cm and had limited stromal invasion (less than 10 mm on conization or less than 50% stromal invasion on imaging). Locally advanced cervical cancer is primarily treated by concurrent chemoradiation and brachytherapy, and distant metastatic disease by (palliative) chemotherapy with carbo-platinum and paclitaxel with or without angiogenesis inhibitor bevacizumab [5,6]. The higher the initial stage of disease, the higher the risk of recurrence, ranging from 10% for stage IB to 74% for stage IV, and most recurrences occur within 24 months of primary treatment [6,7]. Patients treated primarily with chemoradiation and who have residual disease or developed centrally recurrent disease, without distant metastases after a disease-free interval, are candidates for pelvic exenteration with the intention to cure [8,9,10]. Palliative pelvic exenteration in order to remove the tumor burden, to relieve symptoms and to improve (short term) quality of life is debatable and controversial. This procedure has evolved from a palliative to a therapeutic and curative surgical treatment in highly selected cases with centrally located recurrent gynecological cancers, mainly performed in recurrent cervical cancer [11]. Pelvic exenterations include a total en bloc resection of the involved pelvic organs in order to achieve a complete (R0) resection, often with the formation of a urostoma and/or colostomy. The open pelvic exenteration has a high morbidity of more than 50% and is associated with major perioperative and postoperative complications, besides the high psychosocial impact on patients [10]. Therefore, minimally invasive surgery has been introduced for pelvic exenterations in order to decrease the high morbidity of this procedure, without compromising the surgical efficiency and oncological outcomes. Minimally invasive surgery is also associated with shorter hospital stays, increased recovery and better quality of life compared to open surgery. The first published laparoscopic total pelvic exenteration was in 2003 by Pomel et al., and the first reported robotic-assisted total pelvic exenteration was in 2009 by Lim [12,13].

The advantages of robotic-assisted exenterations over laparoscopic exenterations are the observed shorter learning curves of robotic surgery compared to laparoscopic surgery, the enhanced 3D vision at the console, the increased mobility and degree of motion of the wristed instruments, especially advantageous when dissecting in the avascular paravesical and (Okabayashi’s/Latzko’s) medial/lateral pararectal spaces, down into the narrow pelvic floor, when compared to the instruments of conventional laparoscopy [14]. 

Preoperative imaging and tumor board meetings are of utmost importance in selecting suitable candidates for pelvic exenteration in cases of residual or recurrent disease. Besides the clinical performance status (PS) of the patient defined by the ECOG (Eastern Cooperative Oncology Group), the type of pelvic exenteration depends highly on the size of the tumor and the extent of invasion in surrounding tissues/organs [15]. Strict surgical oncological criteria must be applied prior to performing a pelvic exenteration. The tumor should be removed en bloc with free surgical margins, which is a key prognostic factor, and with the avoidance of the dissemination of cancer by manipulation. Positive surgical resection margins are associated with worse survival compared to those with negative margins [8,9]. With carefully selected indications for pelvic exenteration, several studies have reported a 5-year overall survival of up to 64% [11,16,17,18,19,20]. For women with node-negative disease, the median overall survival can be up to 73.2 months [21]. Prospective randomized data comparing the 5-year overall survival between open and minimally invasive pelvic exenterations for recurrent cervical cancer are lacking.

Adequate imaging is essential prior to surgery, both for locoregional disease assessment and for the detection of distant metastases. For locoregional assessment, pelvic magnetic resonance imaging (MRI) has been the preferred imaging technique to determine the tumor size and invasion of surrounding tissues. Recently, MRI-based subdivision of the pelvis into seven compartments has been introduced for planning pelvic exenterations in colorectal cancer patients, reporting high sensitivity for locoregional tumor invasion [22,23]. Positron emission tomography-computed tomography (PET-CT) is the commonly used imaging modality for evaluating the presence of distant metastases. 

In this systematic review, a comprehensive search of studies on robotic-assisted pelvic exenteration is conducted with the primary aim to assess the incidence/rate of early and late postoperative complications as well as oncological outcomes in terms of recurrences and disease-free/overall survival. The findings of these studies are discussed alongside reported incidence rates in open pelvic exenterations in persistent/recurrent cervical cancer. We also discuss the potential application of compartment-based MRI imaging for the preoperative assessment of persistent/recurrent cervical cancer. 

## 2. Materials and Methods

### 2.1. Objectives and PICO Process

The protocol was registered with the International Prospective Register of Systematic Reviews (PROSPERO ID, CRD42024558346). The primary objective of this systematic review is the assessment of early and late postoperative complications, as well as oncological outcomes (disease-free/overall survival) in patients with persistent/recurrent cervical cancer undergoing robotic-assisted pelvic exenteration. The secondary objectives are the type of exenteration, the duration of surgery, blood loss and hospitalization time. 

The PICO criteria are utilized in framing the following research question. In patients with persistent or recurrent cervical cancer (P), does robotic-assisted pelvic exenteration (I) compare to open exenteration (C), result in differences in early and late postoperative complications and oncological outcomes (O)?

### 2.2. Search Strategy

**Figure 1:**
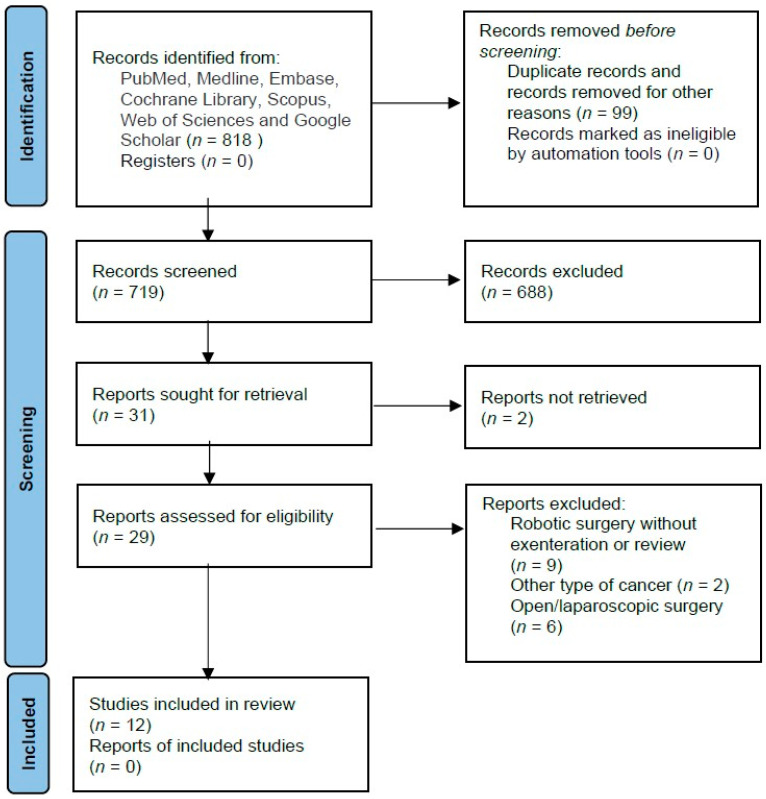
PRISMA flow diagram.

This systematic review adhered to the guidelines outlined in the PRISMA (Preferred Reporting Items for Systematic Reviews and Meta-Analyses) statement for the conduct and reporting of data [24]. The research focused on articles published in peer-reviewed journals. Studies were identified by searching literature databases, including PubMed, Medline, Embase, Cochrane Library, Scopus, Web of Sciences databases and Google Scholar. Using an advanced search strategy, terms such as “robotic” AND “pelvic exenteration” AND “cervical cancer” were employed. Additional screenings of reference lists from previously published articles were conducted (Figure 1).

### 2.3. Inclusion and Exclusion Criteria

Articles published up to and including March 2024 are included for this systematic review. Patients with persistent/recurrent cervical cancer undergoing robotic-assisted pelvic exenteration are included and compared, in terms of differences in early and late postoperative complications and oncological outcomes, with the published rates/incidences for open pelvic exenterations in persistent/recurrent cervical cancer. Studies included are case reports, case series and previously published reviews on robotic-assisted pelvic exenterations in persistent/recurrent cervical cancer. Reports on cervical cancer patients undergoing only a laparoscopic pelvic exenteration or having another type of cancer for pelvic exenteration are excluded.

### 2.4. Data Extraction

Articles on patients with persistent/recurrent cervical cancer undergoing robotic-assisted pelvic exenteration are included. The following data are extracted: the number of patients in each study, the type of robotic-assisted pelvic exenteration, total operative time, estimated blood loss, early postoperative complications, late postoperative complications, hospital stay in days, median follow-up in months, oncological outcome (recurrences, disease-free/overall survival).

### 2.5. Risk of Bias

There are only case reports or case series on robotic-assisted pelvic exenteration for persistent/recurrent cervical cancer available, and this may cause a significant risk of bias in this review. A meta-analysis is not conducted as there are no large retrospective studies nor prospective randomized trials comparing robotic-assisted with open pelvic exenterations.

## 3. Results

The initial number of records identified from several literature databases was 818, of which 99 studies were removed before screening, and 688 were excluded for not meeting the inclusion criteria. As a result, 29 of the 31 reports were retrieved and assessed for eligibility. Finally, our study comprised 12 reports, consisting of four single case reports and eight case series. See Figure 1 and Table 1. 

The type of robotic pelvic exenteration was, in all four case reports, a total pelvic exenteration (TPE). The total operative time varied between 240 and 700 min. Hospital stay was between 9 and 11 days. A fistula occurred as a late postoperative complication in one case. Other postoperative complications were not specified or mentioned. In one case, no recurrence occurred during the 17 months of follow-up; in the other case reports, the oncological outcomes were not specified. 

In six (n = 42 patients) of the eight case series, the type of robotic pelvic exenteration was an anterior pelvic exenteration (APE). In the two other case series, there were seven robotic total pelvic exenterations (TPEs), eight robotic anterior pelvic exenterations (APEs) and two robotic posterior pelvic exenterations (PPEs). The total operative time in these case series varied between 180–540 min. The hospital stay varied between 3–38 days. In the majority of cases, the early and late postoperative complications were specified and are discussed below. The recurrences, disease-free survival and/or overall survival are reported in all case series.

## 4. Discussion

**Figure 2:**
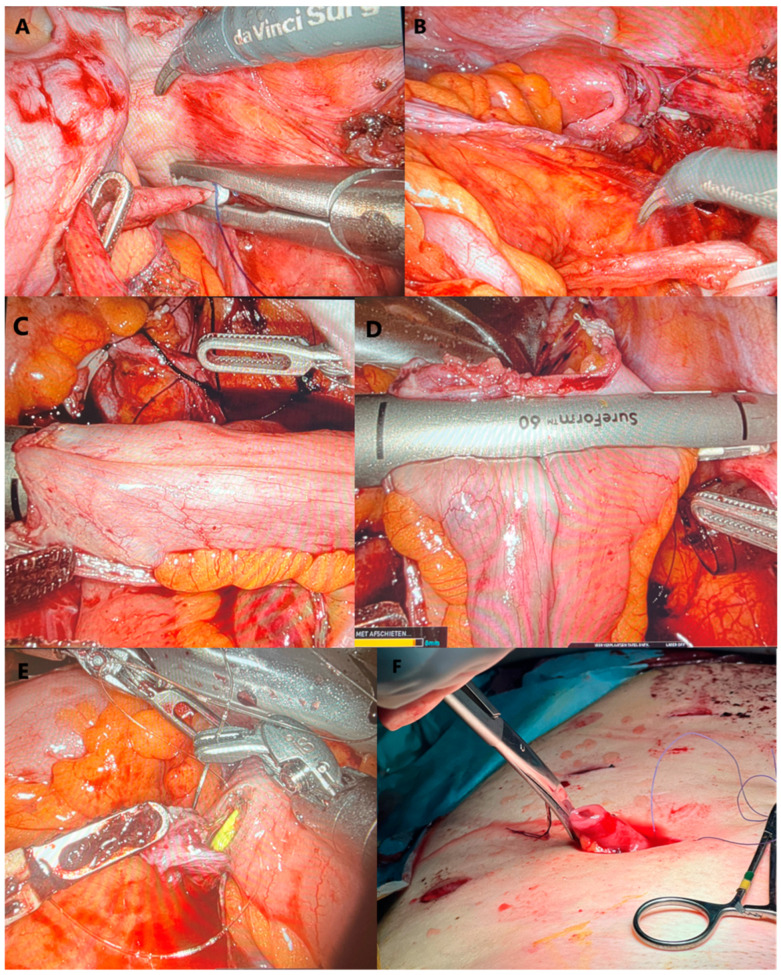
(**A**–**F**). Ileal conduit formation (Bricker’s derivation) during robotic-assisted anterior pelvic exenteration (APE): (**A**) clipping left ureter, (**B**) mobilizing left ureter under meso-sigmoid colon, (**C**) joining two ileal loops with a stapler, (**D**) the closure of the ileal pouch with an Endo GIA 60 mm stapler, (**E**) uretero-ileal anastomosis with the ureter stent in place, (**F**) the urostoma of the ileal conduit.

Pelvic exenteration by open surgery was first described by Brunschwig et al. in 1948, and comprised an ultra-radical surgical procedure involving the en bloc resection of multiple pelvic organs [34]. An anterior pelvic exenteration (APE) involves the complete resection of the bladder, distal ureters, urethra, female pelvic organs (uterus and vagina) and the paracervical and parametrial tissues towards the pelvic side walls. A urinary diversion is often constructed with a ureteral–ileal loop anastomosis and urostoma, the so-called Bricker’s derivation (example in Figure 2A–F). A posterior pelvic exenteration (PPE) includes the resection, besides the female pelvic organs, of the rectum and the mesorectum towards the levator muscles (supralevator exenteration, type I) and the formation of a diverting colostomy. A total pelvic exenteration (TPE) in cervical cancer involves the combination of an APE and a PPE. This open radical procedure with long operative times varying between 5–9 h is associated with early and late complications in 16–71% and 36–61% of cases, respectively, and with potential extensive blood loss ranging from 2.3–4 L; patients are hospitalized between 19 and 37 days [19,30,35]. Hence, minimally invasive surgery for exenterative surgical procedures was introduced to reduce morbidity (with lower perioperative complications) and to improve early recovery after surgery, without compromising the surgical efficiency and oncological outcomes [11]. This is of particular importance given the increase in recent years in obese and comorbid patients.

The first laparoscopic total pelvic exenteration was reported in 2003 by Pomel et al. [12]. The first reported case of robotic-assisted total pelvic exenteration was in 2009 by Lim, and the first cases of robotic-assisted anterior pelvic exenteration were reported by Lambaudie et al. in 2010 [13,25]. Since then, several institutions have published case studies or case series with their experiences in robotic-assisted pelvic exenterations (Table 1).

In 2009, Lim showed, in one case, the feasibility and safety of a robotic-assisted total pelvic exenteration with an ileal loop urinary diversion and colostomy. The total operative time was 375 min; there were no intra- or postoperative complications and there was minimal blood loss. The patient was discharged home after 10 days [13]. In 2010, Lambaudie et al. demonstrated, in three cases, the feasibility of robotic-assisted anterior pelvic exenteration (APE) for recurrent cervical cancer. No intra- or early postoperative complications were observed. The urinary diversions were created with the Miami continent pouch technique using an ileo-colonic reservoir [25]. Also in 2010, Davis et al. published their findings of robotic-assisted anterior pelvic exenteration with ileal conduit formation in two patients with persistent and recurrent cervical cancer [26]. The mean operative time was 9 h and mean blood loss 550 mL, significantly less compared to open pelvic exenterations. Patients were discharged from hospital after 8 days. In 2011, Jauffret et al. reported the application of robotic-assisted surgery in recurrent gynecological cancer [27]. Two patients underwent a robotic-assisted APE for recurrent cervical cancer with a median operative time of 480 min. There were no peri- or early postoperative complications, and there was one late postoperative complication (impaired wound healing). Both patients developed recurrent disease, 8 and 23 months after surgery.

Lawande et al. published, in 2014, a case of advanced cervical cancer involving the bladder and rectum with a rectovaginal fistula and impending vesicovaginal fistula [28]. The patient underwent a robotic-assisted TPE with colo-anal anastomosis and uretero-sigmoidostomy. The total operative time was considerably lower (240 min) compared to open TPE, and with limited blood loss (300 mL). The same institution published, in 2014, their initial experience of robotic-assisted APE in 10 cases of advanced or recurrent cervical cancer [10]. Their mean operative time was 180 min, mean blood loss was 110 mL and mean hospital stay was 5 days. There were no surgically related complications. All patients had a complete (R0) resection with free surgical margins. Eight of the 10 patients were disease-free after a median follow-up of 11 months. Two patients developed metastasis in the liver or para-aortic nodes.

Nguyen Xuan et al. reported, in 2018, the feasibility of robotic-assisted exenteration (anterior, posterior or total) in a case series of six patients with recurrent cervical cancer [30]. The mean operative time was 6.7 h and no major perioperative complications occurred. The surgical margins were free in 67% of cases. Late complications occurred after more than 80 days, on average, and included wound infection, the stenosis of ileal anastomosis, renal failure and pulmonary embolism. There were three recurrences, three patients with pelvic lymph node metastasis, of which one with bone metastasis, occurring within an average of 7 months. A separate case of robotic-assisted TPE was published in 2018, with excellent postoperative results and without any recurrence after 17 months of follow-up [31].

A video-illustrated step-by-step technique of a robotic-assisted TPE was published in 2017 by Konstantinidis et al. in a case with recurrent cervical cancer [29]. 

In 2019, Bizzarri et al. published a case series of 23 patients, with 10 patients having recurrent cervical cancer, and their findings supported the evidence that, in carefully selected patients, laparoscopic or robotic-assisted exenteration is superior to the open approach [19]. Eleven of the 23 patients underwent robotic-assisted exenteration and the overall median operative time was 540 min, with a significantly longer time for the laparoscopic group. Median blood loss was 400 mL and R0 resection was achieved in approximately 75% of cases. The median duration of hospitalization was 10 days, and the median disease-free survival was 11 months. These findings were similar to those reported in a review from the same year [36].

Jain et al. reported, in 2021, their technique and feasibility of robotic-assisted APE for persistent or recurrent cervical cancer in 14 patients [32]. The median operative time was 305 min, the estimated blood loss was 135 mL, and the median length of hospital stay was 6.5 days, which was similar to most previous reports on robotic-assisted APE. Early and late complications occurred in 36% and 28.6% of cases, respectively. All patients had free surgical margins. The 12-month disease-free and overall survival was 68.2% and 77.1%, respectively.

Matsuo et al. reported, in 2021, the data of a retrospective population-based analysis of women with different types of female pelvic cancers who underwent a pelvic exenteration, with a particular focus on the laparoscopic and robotic pelvic exenteration group [11]. Among the 1376 women analyzed, only 3.6% (n = 49) underwent an exenteration via a minimally invasive approach and 51% robotically. The minimally invasive group was more likely to have recurrent cervical or uterine cancers and to receive urinary diversion, but less likely to have vaginal reconstruction or colostomy when compared to the open exenteration group. Perioperative complications were similar; however, major complications including sepsis and thromboembolism were less likely in the minimally invasive group. The hospital stay was also shorter in the minimally invasive group. An overview of the literature in 2021 revealed only 150 cases, studied between 2009 and 2019, who were evaluated for both perioperative complications and oncological outcomes in minimally invasive pelvic exenterations for gynecological cancers [11]. In 78% of the cases, an APE was performed, and the majority had laparoscopic surgery, with 91.7% free surgical margins. The overall intraoperative complications were low (4.7%), and the postoperative complications were, on average, 35.3%. Approximately 50% of the cases were alive at the last follow-up (median follow-up varied from 10 to 44.8 months). In one case–control study by Martinez et al., survival was similar between the open and laparoscopic approach [37]. A systematic review by Cianci et al. in 2021, focusing on robotic-assisted exenterations for different cancer types, revealed 53 cases, of which the majority (79.2%) had an anterior exenteration [17]. The operative times for robotic-assisted APE varied between 180 and 540 min, the mean blood loss was between 110 and 550 mL, and the hospital stay between 5 and 36 days, which are all considerably lower compared to open APE data. The most common and preferred urinary reconstruction was an ileal loop diversion (Bricker’s derivation, example in Figure 2A–F). Free surgical margins were obtained in 88% of cases, and severe postoperative complications occurred in 27.5% of cases (according to the Clavien-Dindo classification: grade ≥ 3). Late postoperative complications were mainly associated with the urinary reconstruction.

In 2024, Dudus et al. reported their experiences in 12 cases of minimally invasive pelvic exenterations for gynecological cancers, of which 83.3% were for cervical cancer [33]. The study included six anterior and six total pelvic exenterations. In total, 9 out of 12 patients underwent robotic-assisted exenteration, with a mean operative time of 360 min for APE and 440 min for TPE. The perioperative morbidity was low (16.6%) and the mean blood loss during surgery was 350 mL. In 75% of cases, a complete (R0) resection was achieved. During follow-up, 50% of patients were still alive 24 months after surgery.

Several case series have shown shorter operative times, lower blood loss, and shorter hospital stay for robotic surgery. In addition, wristed robotic instruments provide an easier suturing of the ureteral–ileal loop anastomosis for the necessary urinary diversion in an anterior pelvic exenteration (APE). In female patients, the robotic-assisted APE is widely accepted in cases of bladder cancer invading the muscular bladder wall [38].

Among the 63 reported cases of robotic-assisted pelvic exenteration, in cervical cancer, 50 (79.4%) were anterior pelvic exenterations. Median operative times ranged from 180 to 700 min, depending on the type (anterior, posterior, or total) of pelvic exenteration; complete R0 resection varied between 67 and 100% of cases. Intraoperative complications were minimal, approximately 5%; early and late major complications averaged between 30 and 35%, markedly lower than those observed with open pelvic exenterations.

## 5. Prognostic Factors

The lymph node status remains controversial in the literature when considering an exenteration. According to some studies, it is associated with worse survival; however, others have not correlated it with an adverse oncological outcome [39,40,41,42,43]. Even pelvic-side-wall recurrences are, in selected cases, no longer an absolute contraindication [18,39,41,42,43,44,45,46]. Therefore, careful individual selection is recommended when considering patients with suspicious lymph node(s) on imaging for exenteration. Several prognostic factors should be evaluated when making a decision on planning a potentially curative pelvic exenteration: the size of the tumor, the disease-free interval between primary treatment, and the occurrence of the recurrent tumor and presence of lymph node metastases at initial presentation [9,18]. Tumors of more than 5 cm have been shown to have the debatable benefit of an exenteration, with a limited-to-no-chance of remission. The 5-year survival rate of patients undergoing an exenteration for recurrent cervical cancer after a disease-free interval of less than 2 years, between 2–5 years and more than 5 years is 16.8%, 28.0% and 83.2%, respectively [9,42]. Postoperatively, positive surgical resection margins are a major prognostic factor associated with decreased survival, with a 2-year survival of 10.2% compared to a 2-year survival of 55.2% for patients with uninvolved margins [9]. 

## 6. Preoperative Imaging for Planning Pelvic Exenteration

### 6.1. Magnetic Resonance Imaging

Magnetic resonance imaging (MRI) is the preferred imaging modality to evaluate the presence of residual or recurrent cervical cancer. The high tissue contrast allows for the precise determination of tumor size as well as the assessment of local tumor infiltration in surrounding tissues/organs (uterine corpus, parametrium, vagina, bladder and rectum). The meta-analysis of MRI studies in detecting recurrent cervical cancer reveals a sensitivity and specificity between 82–100% and 78–100%, respectively [47].

The sensitivity of MRI for detecting invasion in the parametrium, bladder, rectum, and lymph nodes is 74%, 75%, 71% and 60%, respectively. The prediction on the MRI of uninvolved margins, a key prognostic factor for pelvic exenterations, has a sensitivity and specificity of 85% and 52%, respectively [16,48]. The positive and negative predictive values are 60% and 80%, respectively. 

Further improvements of MRI imaging, in order to increase both the sensitivity and specificity for detecting (limited) invasion in surrounding tissues, will enable surgeons to more accurately plan the indication and type of exenteration. An important aspect will be to better distinguish between viable tumor and radiation-induced fibrosis.

### 6.2. Compartment-Based Imaging 

In order to obtain optimal visualization of the complex anatomy of the pelvis for planning major exenterative surgery, seven pelvic compartments have been described on magnetic resonance imaging (MRI), initially for colorectal cancer [23]. These seven compartments are defined as follows: (1) peritoneal reflection compartment, (2) anterior above peritoneal reflection compartment, (3) anterior below peritoneal reflection compartment, (4) central compartment, (5) posterior compartment, (6) lateral compartments, and (7) inferior compartment. Evaluating the accuracy of compartment-based MRI for detecting local tumor involvement, the sensitivity was more than 93% in all compartments apart from the lateral compartment, where it was 89.3%. The specificity for the posterior and anterior compartments below the peritoneal reflection was 82.2% and 86.4%, respectively, which was lower than the other compartments.

In 2023, in Annals of Surgery, the compartment-based anatomy for colorectal cancer was revisited on the basis of MRI studies of 13 pelvic specimens from body donors, of which 6 females, and the anatomical landmarks for each compartment were described in detail [22]. Given that the original and revisited central compartment description for colorectal cancer contains the rectum and mesorectum as well as the pelvic autonomic nerves, the central compartment for persistent or recurrent cervical cancer should contain instead the uterus and cervix with its parametrium, the vagina with its paracolpium, the proximal round ligaments and the uterine/vaginal vessels. 

We have summarized here (Table 2) the other anatomical structures of the different compartments for pelvic exenterations in case of recurrent cervical cancer.

**Figure 3:**
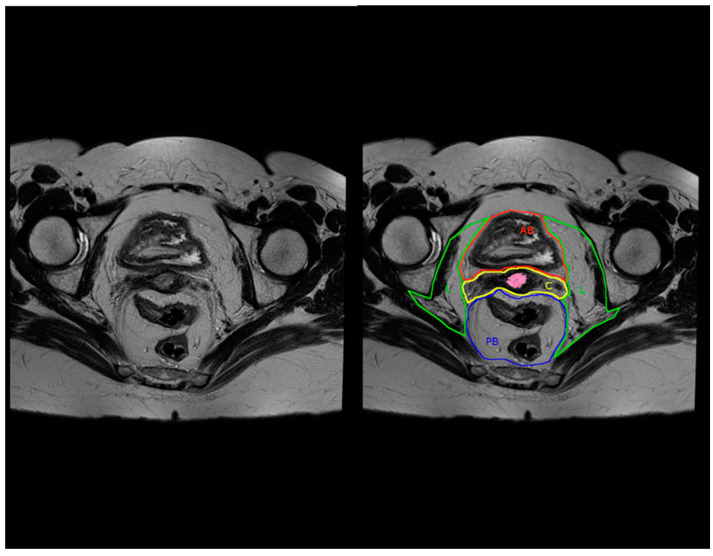
Conventional (**left**) and compartment-based (**right**) magnetic resonance imaging, axial T2-weighted image. AB: anterior below peritoneal reflection compartment, C: central compartment, L: lateral compartment, PB: posterior below peritoneal reflection compartment. Cervical tumor is highlighted in pink.

**Figure 4:**
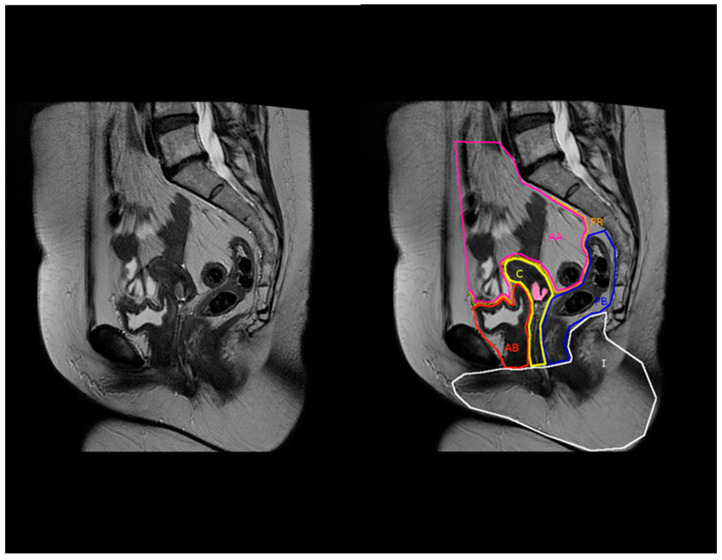
Conventional (**left**) and compartment-based (**right**) magnetic resonance imaging, sagittal T2-weighted image. AA: anterior above peritoneal reflection compartment, PR: peritoneal reflection compartment, AB: anterior below peritoneal reflection compartment, C: central compartment, PB: posterior below peritoneal reflection compartment, I: inferior compartment. Cervical tumor is highlighted in pink and extends towards the anterior below peritoneal reflection compartment (AB).

Applying this compartment-based anatomy with MRI in the preoperative evaluation of recurrent cervical cancers for pelvic exenterations may be helpful in standardizing the selection and type of exenteration, especially given the high sensitivity and specificity of this approach, as mentioned above in colorectal cancer. For example, when a recurrent cervical cancer is located in the central compartment and extends towards or crosses the border with the anterior below peritoneal reflection compartment, without any other pelvic disease, then an anterior pelvic exenteration could be considered (example in Figure 3 and Figure 4).

### 6.3. Positron-Emission Tomography-Computerized Tomography

The preferred imaging modality to evaluate the presence of distant metastases in organs or lymph nodes is positron emission tomography-computed tomography (PET-CT). Chu et al. showed, in 2014, in a systematic review and meta-analysis of 1757 patients, that the sensitivity and specificity of PET-CT in detecting metastases was 90% and 99%, respectively [49]. In 2014, Meads et al. concluded, in a meta-analysis of nine PET-CT studies evaluating the detection and management of recurrent cervical cancer, a sensitivity of 94.8% and a specificity of 86.9% [47].

A more recent study in 2020 on the value of evaluating the presence of para-aortic lymph node metastases in locally advanced cervical cancer by PET-CT versus surgical staging revealed, for PET-CT, a sensitivity and specificity of 23.5% and 93.3%, respectively [50]. The positive predictive value was 47.1% and negative predictive value was 82.8%. A subsequent meta-analysis in 2022 by Thelissen et al. showed, by pooling 12 cohort studies, an upstaging rate of 12% by para-lymph node dissection after a negative PET-CT [51]. When considering only the patients with pelvic nodal metastases on PET-CT without the suspicion of para-aortic lymph node involvement, seven cohorts showed a pooled upstaging rate of 21% by surgical para-aortic staging. 

## 7. Conclusions

In conclusion, robotic-assisted pelvic exenterations have shown to be feasible and safe with reduced operative times, lower blood loss and perioperative as well as postoperative major complications compared to open surgery, while maintaining surgical efficiency. Long-term oncologic outcomes, including survival and recurrence rates, appear comparable between the robotic and conventional open technique, although prospective randomized trials are needed for definitive conclusions. Despite the promising advancements, several challenges remain in the widespread adoption of robotic-assisted pelvic exenterations for cervical cancer, including cost considerations and access to specialized training. MRI and PET-CT are the preferred imaging modalities for evaluating persistent and recurrent cervical cancer to aid decision making when considering pelvic exenteration. The proposed compartment-based anatomy with MRI, with a central compartment containing the uterus and vagina, may be useful in standardizing the selection and type of pelvic exenteration. 

## Figures and Tables

**Table 1:** Robotic-assisted pelvic exenterations in residual or recurrent cervical cancer. Overview of the literature.

Author	Year	Number of Patients	Type of Exenteration	Total Operative Time (mins)	EBL (mL)	Early Postop Complications	Late Postop Complications	Hospital Stay (Days-Range)	Median Follow-Up (Months-Range)	Outcome
Lim [13]	2009	1	TPE	375	375	none	NS	10	NS	NS
Lambaudi et al. [25]	2010	3	APE	480	400	none	Fistula, UTI, ureteral stenosis, perineal abscess	30	2–12	DFS = 9 m
Davis et al. [26]	2010	2	APE	540	550	NS	NS	8	NS	Recurrence after 8 and 23 m
Jauffret et al. [27]	2011	2	APE	480	300	none	Wound dehiscence, fistula, sepsis, urosepsis, prerenal failure, obstructive renal failure	6 (3–24)	23	DFS = 5 m and 8 m, OS = 22 m and 23 m
Lawande et al. [28]	2014	1	TPE	240	300	none	none	11	2	NS
Puntambekar et al. [10]	2014	10	APE	180	110	none	none	5	11	Disease free: 8 patients
Konstantinidis et al. [29]	2017	1	TPE	641	400	NS	NS	NS	NS	NS
Nguyen Xuan et al. [30]	2018	5	APE (n = 2) PPE (n = 2) TPE (n = 1)	390–480	NS	4 UTI, 1 PE, 1 sepsis	Wound infection, stenosis ileal anastomosis, renal failure, pulmonary embolism	11.5	NS	3 recurrences after 7 m, 1 died after 10 m
Yang et al. [31]	2018	1	TPE	700	300	none	Fistula	37	17	no recurrence
Bizzarri et al. [19]	2019	11	APE	500	235	none	27.3% of cases	9	15	DFS = 11 m
Jain et al. [32]	2021	14	APE	305	135	36% of cases: urosepsis, anastomosis leak, ileus, fistula, intestinal obstruction	28.6% of cases: colon perforation, UTI, large bowel obstruction, bleeding, ureteral stricture	6.5	17.5 (10–68)	5 deaths, 12 m DFS = 68.2%, 12 m OS: 77.1%
Dudus et al. [33]	2024	12	APE (n = 6) TPE (n = 6)	360 440	350	Morbidity: 16.6%, urinoma, wound infection	Pyelonephritis, hydronephrosis, thrombophlebitis, renal failure, bowel obstruction, iliac artery fistula	18 (6–38)	24	50% alive, DFS = 12 m, OS = 20 m

APE: anterior pelvic exenteration; PPE: posterior pelvic exenteration; TPE: total pelvic exenteration; mins: minutes; NS: not specified; UTI: urinary tract infection; DFS: disease-free survival; OS: overall survival; PE: pulmonary embolism; m: months.

**Table 2:** Compartment-based magnetic resonance imaging (MRI): anatomical structures of the seven compartments relevant to pelvic exenterations for residual or recurrent cervical cancer.

MRI Compartment Name	Anatomical Structures
Peritoneal reflection compartment (PR)	Peritoneum (covering Douglas and vesico-uterine space
Anterior above peritoneal reflection compartment (AA)	Abdominal cavity with intestines, mesenteric and omental fat, Retroperitoneal space with proximal two thirds of the ureter, ovarian vessels, genitofemoral nerve, iliopsoas muscle
Anterior below peritoneal reflection compartment (AB)	Bladder, urethra, Retzius space, vesico-ureteral junction, superior/inferior vesical vessels, ureter and bladder nerve branches from inferior hypogastric plexus, vesico-uterine ligament
Central compartment (C)	Uterine corpus, uterine cervix, proximal round ligaments, parametrium, vagina, paracolpium, uterine and vaginal vessels
Posterior below peritoneal reflection compartment (PB)	Rectum, mesorectum, splanchnic branches from the superior/inferior hypogastric plexus and hypogastric nerves, rectosigmoid junction
Lateral compartment (L)	Parietal pelvic fascia, inferior hypogastric nerve in meso-ureter, distal third of ureter, iliac vessels, iliac and obturator lymph nodes, sacral nerve plexus, obturator nerve, internal obturator muscle, piriformis muscle, lumbosacral trunk, ovaries, fallopian tubes
Inferior compartment (I)	Anorectal junction, pelvic floor muscles, levator ani muscle, transverse perineal muscle (urogenital diaphragm), perineal body, pudendal vessels and nerves
